# Radon and Neoplasms

**DOI:** 10.3390/toxics11080681

**Published:** 2023-08-08

**Authors:** Marek Andrzej Komorowski

**Affiliations:** Specjalistyczna Praktyka Lekarska Marek Komorowski, 97300 Piotrków Trybunalski, Poland; mafkomorowski@interia.pl

**Keywords:** radon, radon progeny, alpha radiation, prenatal period, carcinogenesis

## Abstract

Radon is a carcinogenic factor, but the effects of the potential carcinogenicity of radon progeny on the human body during the prenatal period have not yet been explored. Based on data regarding the half-lives of radon-222 and radon-220 and their progeny, this paper considers their potential effects on the human body in the prenatal period. Radon-220 represents a small fraction of the total radon concentration in the air, but the dose of radon-220 progeny may have a significant effect in the prenatal period, as the precursors of polonium-212 exhibit substantially longer half-lives than the corresponding precursors of polonium-214. Theoretically, it is possible that radon-220 decay products, particularly polonium-212, are the predominant emitters of alpha particles in the prenatal period. Studies aiming to establish a relationship between exposure to radon during pregnancy and the subsequently observed incidence of childhood neoplasms should consider this observation.

## 1. Introduction

This paper presents a hypothesis regarding the potential effects of very-low-dose ionising radiation of natural origin on human tissues, which are very sensitive to radiation during prenatal development. Even very low doses of radiation can cause cancer. A comprehensive review of the available biological and biophysical data supports a “linear no-threshold” (LNT) risk model—meaning that the risk of cancer proceeds in a linear fashion at lower doses without a threshold [1]. Carcinogenesis caused by low doses of radiation is also a topic of the UNSCEAR report [2].

Environmental radon is the primary natural origin of ionising radiation [3]. Radon is a noble gas that is approximately eight times heavier than air. It is colourless, odourless, non-flammable and soluble in water [4]. Radon is radioactive, and because it is a gas, it can easily penetrate residential buildings through small leaks in floors and accumulate in concentrations exceeding those found in the atmospheric air (especially during the heating season, when this gas is sucked in through small leaks in the floor due to negative pressure in the heated building). As a gas, radon is inhaled and exhaled during the respiratory cycle and is a relatively low-dose radiation source. In contrast, its radioactive progeny (radioisotopes of lead, bismuth and polonium), which are deposited on the epithelium of the respiratory tract, are the main sources of radiation of natural origin. Radon-222, radon-220 and their progeny are found in residential buildings [5,6]. This paper considers the potentially significant contribution of radon-220 decay products to the total dose of radon progeny that the human body receives in the prenatal period. Future observational studies on pregnant women and their children may provide more reliable and accurate insight into the potential relationship between the prenatal dose of ionising radiation of radon decay products and the risk of developing the most common childhood neoplasms.  

## 2. Material and Methods

This paper is based on data regarding the half-life of radon-220 (thoron), radon-222 and their radioactive progeny in the pregnant woman’s body, considering the penetration of radon decay products through the placenta. It evaluates their potential effects on the human body in the prenatal period. In case of radon exposure, we consider the main source of ionising radiation to be composed of alpha particle emitters such as polonium-218, polonium-214, bismuth-212 and polonium-212. Among these, polonium-218 decays mainly in the mother’s lungs due to its relatively short half-life. About 36% of bismuth-212 decays, emitting alpha particles. In addition, the ratio of the polonium-212 (+bismuth-212) to the polonium-214 dose may vary in the foetus’s body compared to the mother, because the polonium-214 precursors have shorter life times than those of polonium-212 and it takes time for the radionuclides to reach the foetus’s tissues. As a result, the ratio of radionuclides from the uranium–radium series compared to the thorium series that could still emit alpha particles in the foetus’s tissues is reduced.

## 3. Results

As a radioactive gas mainly originating from the Earth’s crust, radon penetrates residential buildings through floor leaks. The share of other radon sources, such as building materials, ambient air and water, is smaller [7]. In ventilated rooms, the concentrations of radon decay products are generally lower than in non-ventilated rooms. Time spent in residential spaces is crucial for the radiation dose received, because radon accumulates in buildings. The pandemic lockdown in the years 2020–2022 increased the indoor exposure time by 4% and, consequently, the radiological risk factors by 9% [8]. Equilibrium factor F indicates how many radionuclides occur at a given ventilation level in relation to the state of radioactive equilibrium. F = PAEC (real)/PAEC (virtual): PAEC—potential alpha energy concentration—is measured in nJ/m^3^, PAEC real (measured) depends on the ventilation level and PAEC virtual is the potential alpha energy concentration in a state of full equilibrium [4]. A value of F = 0.4 for radon-222 is usually assumed for residential spaces [7]. The average value of the equilibrium factor of radon-222 and its progeny has been established to be 0.4, but it can vary in range between 0.1 and 0.9 [9]. The wide range of equilibrium factors suggests that location-specific values are more appropriate than worldwide average value [10]. Some measurements of the radon-220 equilibrium factor have also been made: in one such study, an equilibrium factor for radon-220 and its progeny was established for both outdoor and indoor radon-220 (0.004 ± 0.001 outdoors and 0.04 ± 0.01 indoors). The values of the F factor for radon-220 in several published studies are in general agreement with the values reported in this study [11].

Since harmfulness is mainly associated with the impact of radon progeny, it is more accurate to determine the concentrations of radon decay products and, more specifically, of the potential energy of short-lived radon progeny. This is mainly related to the potential energy of the alpha particles emitted by polonium radioisotopes. This method is employed to investigate the degree of exposure to short-lived radon progeny in mining [12]. It allows us to estimate the likelihood of lung cancer development in miners. Alpha radiation can break DNA strands, inducing permanent damage and producing neoplastic transformations [2,7]. Approximate calculations consider only the energy of alpha particles and disregard beta and gamma rays, which entail much lower energies [13]. This paper presents a hypothesis that, in the future, it may become necessary to distinguish between the biological effects of radon-222 progeny and radon-220 progeny. The concentrations of radon-222 decay products are much lower than the concentration of the gas itself because the half-lives of these products are much shorter than the half-life of radon-222 itself. The situation is different with radon-220. Its half-life is about 55 s, so only a portion of the gas that accumulates under a building penetrates through the floor into the building. However, radioactive thorium—another source of radon-220—is also found in building materials from which walls and ceilings are made. The half-life of polonium-216 is very short, but further decay products of radon-220, i.e., bismuth-212 and lead-212, have half-lives of ~1 h and 10.5 h, respectively. Consequently, in the case of radon-220, its indoor concentration can be lower than those of its progeny. Further, in the thorium series, i.e., after lead-212, bismuth-212 and polonium-212 emit alpha particles during decay (for bismuth-212, ~36% is alpha decay and 64% is beta decay). Harmfulness is related mainly to alpha particle emitters, which exhibit much higher energy than the gamma or beta radiation quanta emitted by other radon progeny. Neoplasms represent the stochastic effects of the biological consequences of ionising radiation and, notably, there is no threshold dose for the stochastic effects to occur, but their incidence increases with dose [2,14]. Practically, the energies emitted by gamma or beta quanta associated with bismuth-212 and lead-212 can be disregarded, but these atoms are the precursors of the alpha-particle-emitting polonium-212 (also about 36% of bismuth-212 decays, emitting alpha particles); hence, the greater their concentrations, the greater the ultimate exposure and the more pronounced the risk of the stochastic effects of ionising radiation on the human body. Radon-222 progeny primarily decay in the respiratory tract, as their half-lives are so short that the alpha particles are rapidly released. The influence of natural clearance of the respiratory tract is less important in this case. However, in the case of radon-220 progeny, the precursors of polonium-212 penetrate deeper into the human body and decay in the lungs in relatively smaller quantities than polonium-218 or polonium-214.

Mucociliary clearance in the respiratory tract may occur by shifting mucus particles toward the larger bronchi. Eventually, this upward-shifted mucus can be swallowed, with the radioactive particles absorbed into the gastrointestinal tract. While some radioactive atoms are absorbed into the blood directly from the respiratory tract, others are absorbed by respiratory macrophages. Radon-222 decay products, such as polonium-218, bismuth-214, lead-214 and polonium-214, exhibit short half-lives and a large proportion of these particles decay in the respiratory tract prior to being removed by the clearance mechanism. It is important to note that respiratory clearance does not mean removal of these radionuclides from the body; they remain in the body as they enter other organs via the bloodstream. The air concentration of radon-220 alone is much lower than that of radon-222. However, as far as radon-220 progeny are concerned (i.e., polonium-216, bismuth-212 and lead-212), these elements—apart from polonium-216, whose half-life is approximately 0.158 s—exhibit much longer half-lives (~1 h for bismuth-212 and >10 h for lead-212). Even if the F constant for radon-220 and its progeny is several times smaller than for radon-222 [11], there could still be an accumulation of significant quantities of radon-220 decay products in the air, regarding the potential impact on the human body. It is estimated that the radiation dose of radon-220 progeny is more than an order of magnitude lower than that of radon-222 progeny [15,16]. In Poland, the annual dose of radon-222 and its progeny average at about 1.2 mSv, and that of radon-220 and its progeny at about 0.1 mSv [15]. Significant amounts of radiation from radon-220 progeny were observed in [5], where 55.1% of the total dose originated from radon-222 and its progeny, and 44.9% of the total dose originated from radon-220 and its progeny. Some information about estimated doses from thoron (another term for radon-220, radioactive isotope of radon) can also be found in the UNSCEAR 2019 report; in two different studies it was estimated that thoron contributes 30–35% to the total dose from exposure to radon and thoron [6]. The impact of radon-220 progeny on the respiratory tract is rather insignificant compared to the influence of radon-222 progeny. For the reasons provided above, i.e., the fact that radon-222 progeny decay mainly in the respiratory tract while radon-220 progeny exhibit much longer half-lives, much more time is generally required for the formation of polonium-212 and bismuth-212, so that a greater proportion is absorbed into the blood before it decays in the respiratory tract. When it comes to radon-222 progeny, the share of polonium-210, an alpha particle emitter second to polonium-214, is less significant, as it originates from lead-210, for which the physical half-life is over 20 years and the effective half-life is a few years [13]; the radiation doses of polonium-210 are, therefore, spread over years. The ratio between the quantities of polonium-214 and polonium-212, which are relevant when it comes to the radiation dose received, may change significantly towards the predominance of polonium-212 in a later period after the radionuclides are absorbed into the blood. Although these doses of radiation are very small for adult organisms, their harmfulness in the prenatal period cannot be excluded. Specifically, lead and bismuth easily pass through the placenta and reach the foetus. The primary mechanism for the transplacental transmission of lead is probably simple diffusion. The concentration of lead in the developing baby’s tissue is directly dependent on lead concentration in the umbilical cord blood [17]. However, this process takes time, which changes the ratio between the radon-222 and radon-220 progeny that could still emit alpha particles in favour of the latter. It appears challenging to translate the radiation doses of radon-222 and radon-220 inhaled by a pregnant woman into the doses that ultimately reach the embryo and the foetus. This paper proposes including such a distinction in future studies, for instance, by comparing different ratios of the naturally occurring radionuclides inhaled by pregnant women and their contribution to the different incidence rates of childhood neoplasms that are subsequently observed in different environments (and possibly also congenital defects, embryonic death). While there is much uncertainty in assessing the impact of very low doses of ionising radiation on the human body, the complete repair of DNA damage (especially breaks of both strands—an effect more frequently encountered in alpha radiation) has not been determined for any of the dose ranges tested [18]. Lead has a half-life of around 30 days in the blood; then it diffuses into soft tissues such as kidneys, brain and liver, where it stays longer (for example, the half-life of lead in the brain is 2 to 3 years) [19]; the vast majority of lead accumulates in bones, remaining there for several dozen years [20]. The radionuclides of lead that enter the foetus through the placenta accumulate in its parenchymal organs, and since the foetal life is much shorter than the half-life of lead-210, the effect of this metal can also be neglected here. In turn, lead-212 and lead-214 penetrate the foetal organism and can accumulate in the parenchymal organs. A small amount of radon-222 itself is also absorbed into the lungs and, because it is lipophilic and accumulates in fatty tissue, it may affect the radiation dose received by the foetus too. In the foetal period, the liver and the spleen function as the haematopoietic organs, while the haematopoietic cells divide rapidly, so they are susceptible to ionising radiation. Furthermore, foetal central nervous system cells divide rapidly, particularly at certain stages of foetal life, so these cells are susceptible to ionising radiation. Therefore, there is an obvious convergence between the organs in which lead accumulates—in this case, the radioisotopes of lead that function as the precursors of the alpha-emitting isotopes of polonium—and the organs in which childhood neoplasms are the most likely to develop. A similar observation may apply to bismuth radionuclides, which also function as the precursors of polonium. At this point, account should be taken of the cancer latency period, ranging from a few to several years for leukaemia and several decades for solid tumours [2].  The potential alpha particle energy from polonium radionuclides is released during the foetal period into organs such as the liver, spleen and brain—organs where the most common childhood neoplasms are found to develop, such as leukaemias, lymphomas and brain tumours.

Research on the correlation between exposure to radon (and its progeny) and cancer development has been ongoing for many years. Regarding lung cancer, its association with radon exposure in mine workers was quickly proven [21]. However, for other neoplasms, different results were obtained. For example, in the case of childhood leukaemia, some studies confirmed an association with radon exposure, while in other studies it was not observed. In one such study, the results of the cohort and case-control studies, based on individual data, did not show any significant association between radon exposure and leukaemia risk [22]. Another study’s conclusion was that children born and staying in areas where the risk from ground radon was classified as low were less likely to develop acute lymphatic leukaemia than those born in areas classified as normal and high risk [23]. In a later study, seven case-control studies of childhood leukaemia with the measurement of radon concentrations in the residences of cases and controls gave mixed results, with some indication of a weak association with acute lymphoblastic leukaemia [24]. In another study among 12 ecological studies, 11 reported a positive association between radon levels and elevated frequency of childhood leukaemia, with 8 being significant; several case-control studies on indoor radon exposure and childhood leukaemia were also examined. Most investigations indicated a weak association, with only a few showing statistical significance [25]. On the other hand, in an interesting study from Norway, no association was found for exposure to radon at home and childhood leukaemia, but an elevated nonsignificant risk for cancer in the central nervous system was observed in this study—this association should be interpreted with caution owing to the crude exposure assessment and the possibilities of confounding factors, as the authors state [26]. In all these studies, the impact of radon-222 and radon-220 progeny was not studied separately. If it is polonium-212 that acts as the dominant alpha particle emitter in the prenatal period, it would be advisable to limit the exposure of pregnant women primarily to radon-220 progeny, for instance, by restricting the thorium content in building materials. Preventive measures to reduce exposure to radon-220 progeny are not identical to measures employed to reduce exposure to radon-222 progeny, as these radioisotopes come from two different radioactive series. 

## 4. Discussion

This paper aimed to show that the impact of naturally occurring environmental radon radioisotopes on the human body during prenatal development has been insufficiently explored. The radiation doses considered in the paper are very small, but the LNT (linear no-threshold) model, according to which there is a linear correlation between the radiation dose and increased cancer risk, is widely accepted [1,2,3]. It appears that alpha radiation, with which the highest energies of radiation quanta are associated, may most closely relate to the LNT model. The impact of radon-220 decay products is underestimated in most studies. Radon-222 progeny decay mainly in the lungs of pregnant women, while radon-220 progeny, such as bismuth-212 and lead-212 (the precursors of polonium-212, which decays by emitting alpha particles; 36% of bismuth-212 also decays, emitting alpha particles), exhibit longer half-lives and have more time to penetrate the blood circulation and the placenta, reaching the foetus, before they decay in the lungs or blood of the pregnant woman. Therefore, the ratio of radon-222 to radon-220 progeny may be reversed, and radon-220 progeny that decay and emit alpha particles may become prevalent in the foetus, which should be the subject of further investigation (theoretically speaking, polonium-212 and bismuth-212, but not polonium-214, could be the main alpha particle emitters in the foetus). As these radionuclides also penetrate adult tissues, the question as to how harmful these are is an open question. Even large differences in concentrations of radon-220 products in the air (which may originate in larger quantities from building materials than from the ground) may be accompanied by minor changes in concentrations of total radon (because radon-220 represents a small proportion of the total radon in the air). Alpha particles are released during the decay of polonium-212, bismuth-212 and polonium-214 (polonium-214 may be important in buildings with particularly high radon-222 concentrations). Alpha particles decaying in the liver and spleen of the foetus (which function as hematopoietic organs) explain the particularly high incidence of childhood leukaemia (considering the latency period of leukaemia development). Furthermore, the decay of these nuclides in the central nervous system could explain the occurrence of some central nervous system tumours in children. It may be revealed that even naturally occurring concentrations of radon-220 progeny have a harmful effect during prenatal development. For preventive measures, methods of reducing exposure to radon-222 progeny and 220 progeny must not completely overlap, as radon-220 can originate in larger quantities, for example, from building materials.

## 5. Conclusions

Studies have been conducted to determine whether radon and its progeny could cause neoplasms other than lung cancer, for instance, leukaemia, but their results are ambiguous. This paper draws attention to the need to distinguish the effects of radon-222 progeny from those exerted by radon-220 progeny. During pregnancy, a vast part of the inhaled radon-222 progeny decays in the lungs of pregnant women, due to their relatively short half-lives, which are shorter than the typical respiratory tract clearance time. This is in contrast to radon-220 decay products, which exhibit longer half-lives and have more time to reach the foetus. Conducting observational studies on pregnant women and their children by distinguishing the effects of radon-222 from those exerted by radon-220 appears to be necessary. Radon-220 constitutes a marginal fraction of the total radon accumulating in buildings, but the impact of its progeny on the foetus, as considered in this work, can represent a significant, if not dominant, contribution to the ionising radiation dose received.

## Data Availability

Not applicable.
